# Demand and Supply Side Barriers that Limit the Uptake of Nutrition Services among Pregnant Women from Rural Ethiopia: An Exploratory Qualitative Study

**DOI:** 10.3390/nu10111687

**Published:** 2018-11-05

**Authors:** Afework Mulugeta Bezabih, Mekonnen Haileselassie Wereta, Znabu Hadush Kahsay, Zewditu Getahun, Alessandra N. Bazzano

**Affiliations:** 1 Department of Nutrition and Dietetics, School of Public Health, College of Health Sciences, Mekelle University, P. O. Box 1871, Mekelle, Ethiopia; afework.mulugeta@gmail.com (A.M.B.); hadiszinabu@gmail.com (Z.H.K.); 2 UNICEF Head Office, P. O. Box 1169, Addis Ababa, Ethiopia; zgetahun@unicef.org; 3 Tulane University School of Public Health, Department of Global Community Health and Behavioral Sciences, New Orleans, LA 70125, USA; abazzano@tulane.edu

**Keywords:** demand, supply, barriers, maternal nutrition, pregnancy, Ethiopia, health services

## Abstract

Despite poverty reduction and increased promotion of improved nutrition practices in the community, undernutrition in Ethiopia remains a concern. The present study aimed to explore the demand and supply side barriers that limit the uptake of nutrition services among pregnant women from the rural communities of the Tigray Region, Northern Ethiopia. A community-based qualitative study was conducted in December through January 2017. A total of 90 key informant in-depth interviews and 14 focus group discussions were undertaken. Study participants were purposively selected for specific characteristics, along with health professionals deployed at various levels of the health system, including health posts, health centers, woreda health offices, and the regional health bureau. Study participants were asked to identify the barriers and implementation challenges that limit access to nutrition services for pregnant women. Participants’ responses were transcribed verbatim, without editing the grammar, to avoid losing meaning. The data were imported to ATLAS.ti 7 (qualitative data analysis software) for coding and analyzed using a thematic content analysis approach. The study findings indicated that the dietary quality of pregnant women in the study area remains poor and in some cases, poorer quality than pre-pregnancy. Across study sites, heavy workloads, food taboos and avoidances, low husband support, lack of economic resources, lack of awareness, low educational level of women, poor dietary habits, increased expenditure for cultural and religious festivities, “dependency syndrome”, low physical access to health facilities, poorly equipped health facilities, focus on child health and nutrition, poor coordination among nutrition specific and sensitive sectors, and limited sources of nutrition information were identified as the demand and supply side barriers limiting the uptake of nutrition services during pregnancy. In conclusion, the community would benefit from improved social behavior change communication on nutrition during pregnancy and multi-sectoral coordination among nutrition-specific and nutrition-sensitive sectors.

## 1. Introduction 

Appropriate nutrition during pregnancy is key to the wellbeing of both the mother and child [1]. Undernutrition among pregnant women is also clearly linked to negative maternal health outcomes, including the risk of maternal mortality and negative pregnancy outcomes [2]. The most sensitive measure of acute nutritional stress during pregnancy is the lack of maternal weight gain [3]. Adequate weight gain during pregnancy is important for optimal fetal growth and development and for maternal fat store deposits [4]. Research indicates the outcomes associated with undernutrition during pregnancy include low birth weight, preterm birth, micronutrient deficiencies, low nutrient stores in infants, fetal growth restriction, perinatal mortality, child stunting, later adulthood chronic diseases, and maternal mortality [5,6]. Furthermore, one quarter of all newborn deaths related to undernutrition may be linked to poor maternal nutrition during pregnancy and fetal undernutrition [5,7]. Moreover, anemia during pregnancy has also been identified as an underlying cause in approximately 20% of maternal deaths and is a key contributor to short stature (stunting) [8], a consequence of chronic undernutrition during early childhood [9]. Thus, malnutrition during pregnancy has far-reaching consequences as it can serve as a fertile ground for perpetuating the intergenerational cycle of malnutrition in women and their newborn children [10,11].

Undernutrition during pregnancy has remained one of the serious challenges in Ethiopian mothers. Nearly 30% of pregnant women from Western Ethiopia were reported to be undernourished (mid upper arm circumference less than or equal to 21 cm) [12]. Similarly, a study conducted on 217 pregnant women attending antenatal care in Butajira General Hospital of Southern Ethiopia reported that 27.6% of them had anemia [13]. A study on weight gain during pregnancy in pregnant women from the Harari Region, Eastern Ethiopia, revealed that about 70% of the pregnant women had inadequate weight gain during their pregnancy [14]. Inadequate intake of folic acid through the diet or lack of adherence, wrong timing of the intake of folic acid supplements, poor antenatal attendance, taste and side-effects of supplements, and challenges with the uninterrupted micronutrient supplies (logistical issues) during pregnancy were the main reasons for the generally poor coverage of supplement intake in pregnant women from low- and middle-income countries, including Ethiopia [15]. Moreover, small proportions of women (as low as 2%) took iron-folic acid supplements at a protective period against neural tube defects [16]. Recent reports from Northwest Ethiopia reported that about 55% of pregnant women adhered to the recommended iron and folic acid supplementation [17]. A recent study from three teaching hospitals in Addis Ababa, Ethiopia, reported 126 neural tube defects (NTDs) per 10,000 births after 12 weeks of gestation [18].

Dietary diversity during pregnancy has been reported to be associated with a reduced risk of under-nutrition in pregnant women from rural Ethiopia [19]. However, pregnant women are still suffering from household food insecurity and low dietary diversity. About 43% of pregnant women from Gambella, Western Ethiopia, were food insecure households and 40% had low diet diversity score [12]. These, along with inadequate obstetric care, contribute to high rates of maternal mortality and poor birth outcomes. Considering the high impact of nutrition during pregnancy in improving women’s own and their newborn’s health, reducing child stunting and breaking the intergenerational cycle of malnutrition in women and their newborn children, the right investment to improve nutrition during conception is an issue that deserves due attention and action in the Ethiopian setting.

Hence, Ethiopia needs to accelerate its efforts to reach the second National Nutrition Program (NNP II) target of reducing the prevalence of anemia among pregnant women from 22% to 14% and reduce the proportion of newborns with low birth weight (less than 2.5 kg at birth) from 11% to 5% by 2020 [20]. Similarly, the second Health Sector Transformation Plan (HSTP II) aims to significantly improve the proportion of pregnant women supplemented with iron folic acid from 19% in the base year (2015/2016) to 100% in 2019/2020 [21]. However, these ambitious targets can be achieved if the context-specific demand and supply side barriers to maternal nutrition during pregnancy are identified and interventions are designed accordingly. Demand-side barriers are defined as “individual, household or community characteristics that influence the demand for nutrition services” whereas supply side barriers are “those characteristics of the health system that exists beyond the control of potential health service users, such as health facilities, drugs, equipment, finances, human resources, geographic distance, and so on” [22]. Thus, the objective of this study was to explore the demand and supply side barriers that limit the uptake of nutrition services during pregnancy among pregnant women from rural communities of Ethiopia.

## 2. Methods and Materials

### 2.1. Study Setting

**Figure 1:**
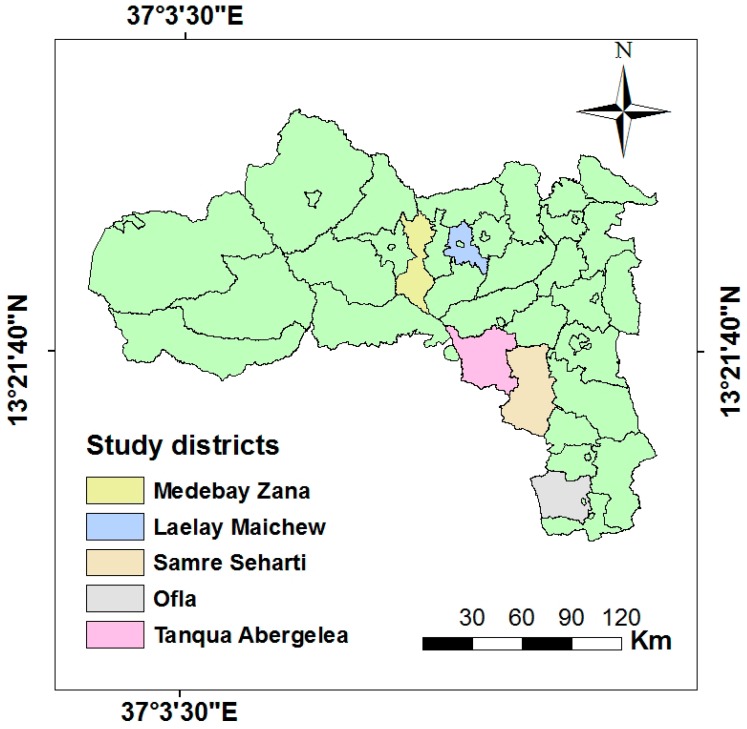
Study districts from the Tigray Administrative region, Northern Ethiopia.

The study was conducted in three food-insecure districts of the Tigray region, namely Ofla, Samre Seharti, and Tanqua Abergeleà, located along the Tekeze basin and two food-secure districts, namely Laelay Maichew and Medebay Zana situated outside the Tekeze Basin (Figure 1). Farming and animal husbandry remain the main stays of the local economy of the study areas. The three food-insecure districts are intervention areas of the Seqota Declaration, a commitment of the Ethiopian Government to end childhood under-nutrition by 2030 [23]. The two food secure districts were included for comparative purposes. Data was collected from both the food insecure and secure districts from November 2017 through January 2018. A district is considered food insecure if it incorporates low food intake, variable access to food, and vulnerable to a shortage of food or shocks [24]. Similarly, a household is considered chronically food insecure if it has been a recipient of food aid for a significant period, generally for the last three consecutive years [25].

### 2.2. Study Design

A community-based exploratory qualitative study was conducted to explore the demand and supply side-barriers affecting the utilization of nutrition services among rural pregnant women in Northern Ethiopia.

### 2.3. Sample

Participants in focus group discussions included pregnant women, husbands, and Women Development Group/Army (WDG/A) leaders. The focus group discussions (FGDs) were held separately with each group to maintain homogeneity in the groups. The WDGs are part of the women’s association and composed of 25–30 women over the age of 18; they include women in neighbouring households who volunteer to organize “1-to-5 networks” [26]. One woman is democratically elected to be the leader of the “1-to-5 network” (Tigrigna: hade lehamushte). The WDGs are meant to create a demand for healthcare, wellness, and improved access to health care services. The system is conceptualized as a way to community ownership of health and is considered critical to the successful implementation of the health extension program (HEP) in Ethiopia [9,26]. In addition, administrative and professional officers ranging from kebele to regional level in governmental and non-governmental sectors were recruited for key informant interviews (KIIs). The governmental sectors included were health, education, agriculture, water resources, and women affairs. Purposive sampling was used to select study participants based on maximum variation. The participants were selected from the study areas by health extension workers considering their experiences and knowledge on the barriers that limit the utilization of nutrition services for pregnant women. Pregnant women who were healthy and without chronic diseases during the period of data collection were included and those who refused to participate and missed their first appointment were excluded from the study. Each FGD constituted 8–10 participants. The study districts were randomly selected. However, the two tabias or kebelles from each district were purposely selected. A tabia or kebelle is the smallest administrative unit comprising of about 5,000 people [27].

### 2.4. Data Collection Tools

Semi-structured discussion and interview guides were developed by a team consisting of experts from multi-disciplinary areas, namely public health, agriculture, nutrition, and food science and technology. The guides covered existing nutrition platforms for nutrition services and barriers to the uptake of nutrition services during pregnancy (Table S1). The guides were pretested before use in tabias of similar setting to the study tabias. Moreover, the guides were reviewed for any adjustments based on feedback from debriefing sessions on a daily basis during the data collection period.

FGDs were held in either the village’s health post or community development centers where privacy and low ambient noise for recording were optimal. Discussions were conducted in a circular seating pattern where participants could engage face to face. For each FGD, a moderator and a note taker were assigned. Data were audiotaped, de-identified, and transferred to an encrypted personal computer for security and confidentiality. The audio data from FGDs and KIIs were transcribed verbatim to the local language (Tigrigna) and then the transcribed data was translated into English. Each transcribed data was assigned a unique file name and saved on a personal computer.

### 2.5. Data Analysis

The translated transcripts of the in-depth interviews (IDIs) and FGDs were imported into ATLAS.ti version 7 qualitative data analysis software. The transcribed data were openly coded by the research group. There were only two coders and coding was done independently. The agreement between the two coders (inter-rater reliability) was determined through continuous discussion between the two coders. Though not encountered, disagreements were settled in consultation with a qualitative data analysis expert. The principal investigator reviewed all coding and ensured cohesion in the approach and use of themes. Categories and themes were identified based on meticulous and systematic reading and coding of the transcripts. Themes with similar domains were then classified based on the study objective and the data collected [28]. Particular attention was given to how many participants shared a certain idea in some of the quotations to guide the trustworthiness of the data. Participants’ quotes were reported directly as they were spoken, without editing the grammar to avoid losing meaning [29].

### 2.6. Ethical Consideration

The study was approved by the Institutional Review Board (IRB) of Mekelle University (Reference number: ERC 1113/2017) and permission to conduct the study was obtained from the Tigray Regional State Health Bureau. Before the start of any data collection, participants were informed about their right to participate and refuse, as well as the purpose of the study and confidentiality of the information provided. Following this, verbal consent to participate was sought from each FGD and KII participants.

## 3. Results

### 3.1. Characteristics of Study Participants

A total of 90 KIIs and 14 FGDs were conducted. The participants represented a wide range of age (22–78 years) and occupation. The durations of FGD and KII range between 70–90 and 45–70 min, respectively. Most of the FGD participants were without formal education or with a low level of educational attainment (Table 1).

### 3.2. Emergent Themes

**Figure 2:**
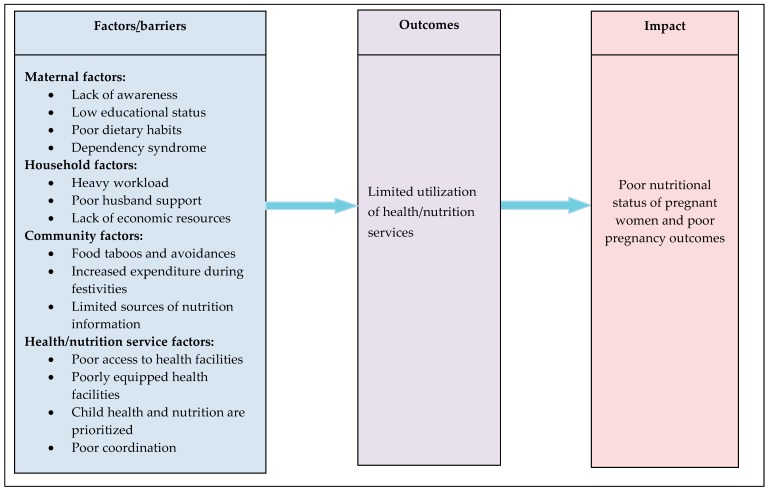
Conceptual framework of factors/barriers limiting the uptake of nutrition services during pregnancy among women from rural communities of Tigray, Northern Ethiopia, 2017/18.

Various themes were identified around the demand and supply side barriers to access nutrition services among pregnant women in the study area. Maternal factors such as lack of awareness, low educational status, poor dietary habits, dependency syndrome; household factors such as heavy workload, poor husband support, lack of economic resources; community factors such as food taboos and avoidances, increased expenditure for cultural and religious festivities, limited sources of nutrition information; and health/nutrition service factors such as poor access to health facilities, poorly equipped health facilities, focus on child health and nutrition, poor coordination among nutrition specific and sensitive sectors were identified, as shown in Figure 2.

### 3.3. Maternal Factors

#### 3.3.1. Lack of Awareness

The health extension workers provide nutrition counseling on various nutrition services such as iron folic acid supplementation, use of iodized salt, utilization of insecticide treated bed nets, antenatal care (ANC) follow up, and others. However, the implementation and uptake of the nutrition services are compromised in the study communities. KII and FGD participants indicated that:

Despite the repeated counseling and demonstration sessions, women poorly practiced the health education messages at home. For example, fish which is the most nutritious food and can be harvested from the nearby lake called “Hashenge Lake” is not being utilized by the surrounding communities due to perceived bad smell and poor awareness of the nutritional benefits of eating sea foods (KII, male, age 38 years).

Eye diseases such as night blindness are common during the early days of the rainy season as a result of evaporation of water from the ground (KII, female, age 37 years).

The cause of night blindness in pregnant women is lack of butter (natural oil) to moisturize and nourish the skin of the skull and the hair. Natural butter protects against the dryness of the skull and makes the skull smoother and healthier and eventually prevents eye diseases. Thus, pregnant women should use butter for their skull and hair to prevent eye diseases such as nigh blindness during pregnancy (FGD, male, 40 years).

Drinking water from some water sources such as “Adewta in Hugumbrda Kebelle” is the main cause of goiter during pregnancy (KII, female, age 30 years).

#### 3.3.2. Low Educational Status

Obvious differences were observed between educated and non-educated mothers regarding the utilization of nutrition services, as indicated in the following transcript.

Education is essential to utilize nutrition services. Those mothers who got married after they complete their education are early adopters or good at the utilization of nutrition services. However, mothers without education are resistant to utilize the nutrition and health services (KII, woman, age 32 years).

#### 3.3.3. Poor Dietary Habits

Poor dietary habits and perceived cultural and religious beliefs of women during pregnancy, especially in the rural areas, were identified as barriers to the uptake of nutrition services. A KII indicated that feeding habits might impact the uptake of nutrition services.

Amidst the availability of diversified foods such as cereals, pulses, vegetables, fruits, and animal source foods in the households, there are women who entirely depend on a monotonous diet composed of injera (a soft pancake made from a cereal crop called Eragrostis Teff) and shiro (a sauce made from legumes). The nutrient dense foods are destined for market sales and the money earned is used to purchase foods of low nutrient density such as coffee, sugar, and others (KII, male, age 47 years).

There are also perceived religious or cultural beliefs that affect the dietary habit of pregnant mothers in the study areas. Pregnant women avoid animal source foods during fasting periods. A KII participant reported that:

During fasting periods, orthodox pregnant and lactating women are not allowed to eat foods of animal sources and are expected to stay fasting till mid-day and beyond which might have a significant nutritional impact on themselves and fetuses (KII, woman, age 26 years).

#### 3.3.4. Dependency Syndrome

Majority of the respondents reported that the dependency of people on cash aid or safety net programs is becoming a serious concern to the community. The idea of using a safety net program to come out of poverty and become self-reliant or food secure household is misunderstood by the community and compromises the implementation of nutrition interventions or utilization of nutrition services. A KII participant from Medebay Zana stated that:

The attendance rate for a training that requires the relocation of the trainee is very high as the relocation is compensated with a daily subsistence allowance. However, the same training that does not require the relocation of the trainees from their surroundings is not attended as their participation is not compensated. Thus, the small amount of money is the driving force to participate in the training and implement the intervention than the benefits of the training to the pregnant women (KII, woman, 44 years).

### 3.4. Household Factors

#### 3.4.1. Heavy Workload

Pregnant mothers were often unable to adhere to appropriate healthy nutritional behaviors largely due to high household and field workloads. The participant women reported that they felt hungry, tired, and weak throughout their pregnancies. The increased workload and decreased nutritional support compromise the women’s abilities to maintain the energy level necessary to perform activities of daily living. A majority of the pregnant women interviewed described difficult environments (hot climate and corrugate landscape to access nutrition services) and increased workload as being detrimental to a healthy pregnancy.

An FGD participant from Laelay Maichew, a food secure district, reported that:

Since the length of days during the autumn is short, we are forced to work including the nights. Thus we have no time to prepare and consume diversified foods although we produce different foods types such as teff, wheat, barley, kidney bean, lentil, egg, milk, and vegetables. (FGD, pregnant woman, age 22 years).

Some of the FGD and KII participants reported that pregnant women are engaged in longer working hours than their husbands, who were not necessarily fully occupied in their tasks.

Since they are farmers, they don’t have time to get rest. They weed, harvest, collect, and trash, the agricultural products equally with their husbands and they also do the house work activities. The introduction of simple agricultural technologies should be encouraged at the household level and the families should share the workloads of the pregnant women (KII, male age 52 years).

Similarly, most of the FGD and KII participants reported that pregnant women reduced their food intake on various occasions mainly due to the high agricultural workload and housework activities.

#### 3.4.2. Poor Husband Support

The FGD participants stated that more attention is given to child health and nutrition than to pregnant women by both the government and husbands. An FGD participant woman described the situation as follows:

Women from rural areas are systematically deprived of certain rights. Government employed women in urban areas are getting maternity leave and they will have adequate time to rest and care for their children. But, mothers in rural areas are not getting enough support from the government and the family to lessen their workload suggesting that workload during pregnancy and the health and nutrition of pregnant women are not priorities for the government and the families (FGD, pregnant women age 32 years).

Some discussants stated that pregnancy and childbirth are viewed primarily as women’s responsibilities and hence men have limited involvement. This cultural attitude was reflected by most of the women describing that they are on their own during pregnancy and received little assistance from their husbands. Households are reported to be dominated by husbands, who are not very aware of the support needed by women during pregnancy.

Some husbands still prevent pregnant women from visiting health centers for the sake of their advantage. They assume that she could stay long in the health center and they might be obliged to prepare and serve food for themselves (KII, woman, age 26 years).

A KII participant added that:

There is unfairness in the household. Nobody listens to the ideas from mothers. There is no respect for mothers. The father will dominate his wife and he will not accept whatever golden idea is raised by his wife (KII, woman, 35 years).

On the other hand, there were respondents who reported that husbands are slowly changing their patriarchal traditions and helping pregnant women with household activities. Some women did speak of having supportive husbands, who helped them during pregnancy and made the experience easier, as explained in the following quote:

Husbands of pregnant women are involved in fetching water, washing clothes, collecting woods, providing emotional support, calling an ambulance, marketing and purchasing additional foods from the local market (FGD, pregnant woman, age 30 years).

Regarding the improvement of the husband’s support, participants mentioned that:

Husbands are being counseled through different mechanisms like peer to peer training, experience sharing among themselves, preparation of award for those who treat their pregnant women with respect, getting training from their school students, and repeated training by the experts (KII, male, age 35 years).

#### 3.4.3. Lack of Economic Resources

The respondents indicated that the lack of adequate food and financial resources affected women’s abilities to follow healthy nutritional practices in their communities. According to a majority of the participants, dietary diversity is directly linked to their economic status.

Improved maternal nutrition during pregnancy is all about income. Awareness alone is not going to help. If I don’t have the money, where can I get the additional food? I am always willing to eat extra meals and diversified food during my pregnancy. It is the shortage of income that mostly forces me to share the available foods with my family members (KII, woman, age 26 years).

An FGD participant added that:

Lack of a balanced diet is a serious problem for pregnant women especially during July and August, the rainy season. My wife is thin and our children are thin too. Due to household food insecurity, we are forced to reduce the quantity of the food to our children which ultimately has led to thinness and underweight in my children (FGD, male, age 42 years).

### 3.5. Community Factors

#### 3.5.1. Food Taboos and Avoidances

Both women and men from the tabia level reported that food taboos are commonly practiced in the communities. A male participant advised that pregnant women should be careful and avoid certain substances and foods during their pregnancy.

The community strongly believes that what a woman eats during her pregnancy goes directly to the womb to feed the baby. Thus, some foods can hurt or affect the fetus like “Humodiya” (a local term for a crop disease). It is totally forbidden for pregnant women to go to an agricultural field where the crops are affected by “Humodiya” as it is believed to even cause abortion (FGD, male 35 years).

A KII participant from Lemlem kebelle added that:

Roasted chickpea and roasted wheat are considered as food taboos during pregnancy. If a pregnant woman eats these foods, her brain will be affected and her labor will be painful (KII, woman, age 42 years).

The food taboos or foods avoided during pregnancy in the study communities are indicated in Table 2.

Divergent opinions were noted regarding food taboos during pregnancy in the study communities. Most of the pregnant women indicated that food taboos during pregnancy are not common in their localities. Women believed that no pregnant woman should avoid food, except during illness. A KII participant from the region indicated that:

Except for few women without education and who live in the remote rural areas, I do not think that many still believe foods need to be avoided during pregnancy (KII, male, age 52 years).

#### 3.5.2. Increased Expenditure on Cultural and Religious Festivities

Nearly every respondent noted that agricultural product management and consumption of diversified foods in the households was challenging. Most of their agricultural food products were exhausted within three to four months after harvest due to increased and costly festivities. One KII indicated that increased expenditures associated with cultural or religious festivities are exposing households to food shortages.

Unplanned expenditures on cultural and traditional festivities have led the communities to food shortages and hence nutritional problems to the most vulnerable segments of the population particularly mothers and their children (KII, male, age 35 years). 

#### 3.5.3. Limited Sources of Nutrition Information

The main sources of nutritional information for pregnant women are health extension workers, women development groups, health experts and tabia/kebelle leaders. However, participants expressed concerns regarding the coverage and quality of the information provided. An FGD participant expressed her feelings as follows:

The tabia/kebelle is very vast and hence the information about nutritional improvement in pregnant mothers couldn’t cover all households. Moreover, the information itself is more general and not focused. There is no any specific information on the amount and type of foods important to the pregnant women (FGD, pregnant woman, age 38 years).

### 3.6. Health/Nutrition Service Factors

#### 3.6.1. Poor Access to Health Facilities

Amidst the expansion of health facilities, physical access to these facilities is still a challenge due to long travel distance, lack of road access or transport services, and lack of ambulances. Most of the respondents described that motor vehicles (especially cars) face road access problems and distances to health facilities and public markets in the study area are long, as one FGD participant stated:

The minimum travel distance from one kebelle to the other is twenty-three kilometers. This makes cumbersome for the mothers to visit the health facilities for follow up, advice and training. My sister’s daughter died due to postpartum hemorrhage before five years. She had to deliver at home as there was no road access to her residence (FGD, pregnant woman, age 38 years).

#### 3.6.2. Poorly Equipped Health Facilities

The absence of drugs and supplies and frequent blackout/outage of electricity services in the health facilities were also mentioned as barriers to the proper utilization of health and nutrition services in the communities. An FGD participant claimed that the unavailability of basic amenities were the main reasons for delayed health services.

Due to a shortage of supplies, pregnant women who came to the health facilities after a long travel distance are given a second appointment. These women will not go back to the health facility as they think that the problems still exist (FGD, male, age 31 years).

#### 3.6.3. Focus on Child Health and Nutrition

The respondents indicated that health or nutrition education services focus on child feeding and vaccination. Mothers are sustainably educated to deliver their babies at the health facility and feed their child appropriately, but they are not counseled on their own nutrition. This was consistent across the study districts. The women development groups and health extension workers focus on general issues like health facility follow-up, environmental health, and child feeding. The lack of concern for maternal nutrition from the local administrators and fast turnover of the health workers due to harsh environmental conditions were also mentioned as serious challenges to the implementation of nutrition interventions during pregnancy and lactation.

Since the weather condition of the area is very hot, no expert is interested to stay here for long. The staff turnover is very high. In such a situation, the transfer of different activities and documents are not properly done and hence services are compromised (KII, male, age 25 years).

#### 3.6.4. Poor Coordination

Communities have the resources to address the nutritional problems of children and mothers. However, the relevant sectors are not working together to optimally utilize the available resources to address nutritional problems of women and children. Respondents asserted that due to poor collaboration or coordination among the nutrition-sensitive and nutrition-specific sectors, the resources of the communities are not properly utilized to address malnutrition, as explained in the following transcript:

Fish harvested from the Hashenge Lake is not being utilized due to poor coordination between the nutrition sensitive and nutrition specific sectors such as agriculture, health, water, media and education sectors (KII, male, age 45 years). 

## 4. Discussion

Maternal factors (such as lack of awareness, low educational status, poor dietary habits, dependency syndrome); household factors (such as heavy workload, poor husband support, lack of economic resources); community factors (such as food taboos and avoidances, increased expenditure for cultural and religious festivities, limited sources of nutrition information); and health/nutrition service factors (such as poor access to health facilities, poorly equipped health facilities, focus on child health and nutrition, poor coordination among nutrition-specific and sensitive sectors) were identified as the demand and supply barriers that limit the uptake of nutrition services during pregnancy.

Low awareness was identified as a barrier limiting the uptake of nutrition services in the study communities. This result is similar to a study conducted in other regions of Ethiopia, which demonstrated that better nutritional awareness and nutritional information during pregnancy were identified as important predictors of knowledge of women during pregnancy [30]. A quantitative research study conducted in Bangladesh [31] also found that medium and high maternal knowledge was strongly associated with higher odds of consuming ≥ 5 food groups, compared to women with low knowledge. The limited nutritional knowledge and low awareness found in this study may be explained by a lack of nutrition-related information and counseling. In line with our findings, another study found that nutrition education intervention had a positive effect on the nutritional awareness of pregnant women [32]. A study on the effect of nutrition education on dietary awareness and practice showed that pregnant women in intervention who consumed a daily minimum of three meals or more had a weight gain of 2.1 kg and showed behavioral change, as compared to the non-intervention group [33].

The participants reported that the educational level of a pregnant woman is believed to help improve the utilization of nutrition services, compared to uneducated pregnant women. This is in line with a quantitative study conducted in other regions of Ethiopia [30], which indicated that relative to women with no education, women with primary education had significantly greater odds of nutritional knowledge during pregnancy (Adjusted Odds Ratio=3.09, 95% Confidence Interval: 1.63–5.86). Similar findings were observed between educational status and nutritional awareness in pregnant women (*p* < 0.001) from Islamabad, Pakistan [34]. The findings from a retrospective cohort study from China also reported that prenatal care, in combination with maternal educational level, had a synergetic effect on better pregnancy outcomes [35].

Maternal dietary practice during pregnancy is thought to be one of the factors influencing the health of both the mother and her growing fetus [1]. Current findings suggest that poor dietary practices of women during pregnancy were identified as barriers to the uptake of nutrition services. The diet of pregnant women was predominantly composed of cereals and pulses, consumed in the form of injera and legume-based stews. A recent quantitative study from Ethiopia indicated that 203 (33%) study participants avoided certain foods, of whom about 61.7% skipped their usual meal—the most commonly skipped meal was breakfast [36,37]. Similarly, about 61% of pregnant women from Ethiopia were reported to have poor dietary practices during pregnancy [37]. A change in nutritional habits during pregnancy was reported from Ankara, Turkey, where the consumption of fruits and vegetables had increased, but the intake of fish and red meat decreased during pregnancy [38].

There is concern that once people are accustomed to receiving free food, they will be less willing to make contributions to community development projects that could improve the nutrition situation of the communities without being paid [39]. In the present study, most respondents reported that they need help in terms of money or food from the government and non-governmental organizations, despite their relative economic position. The community has been consistently receiving small amounts of money and food mainly from non-governmental organizations; as a result, they may develop a dependency that undermines nutritional self-efficacy. Programs such as the productive safety net program (PSNP) of Ethiopia, characterized by the engagement of households with able-bodied adult labor, have resulted in improved child nutrition [40] and eventually maternal nutrition. During periods of reduced food supply, mothers are likely to reduce their own intake to secure those of infants and small children [41]. Therefore, moving from free food distributions/aid to public work schemes and from relief provision to more developmental approaches might be vital to improving maternal nutrition during pregnancy.

An increased workload compromises a pregnant woman’s ability to maintain the energy level necessary to perform activities of daily living [42]. A heavy workload for women might lead to poorer diets, not only for their children and other members of the families, but also for the women themselves [43]. Most of the FGD and KII participants reported that pregnant women do an equal amount of work in agricultural production activities with their husbands, in addition to household responsibilities such as collecting firewood and water, taking care of children, and food preparation. The participants agreed that pregnant women’s daily activities were too strenuous and that they could not get sufficient rest even as they approached their delivery date. They reportedly worked from the time they woke up until the time they went to sleep and were physically and emotionally exhausted. This is consistent with studies conducted in Tanzania and rural Gambia [42,44], which reported that the physical and emotional work burden and the lack of rest throughout the duration of pregnancy was a commonly raised issue in most of their study participants.

Research from several countries suggests that a husband’s support during pregnancy improves delivery outcomes, such as significant reductions in length of labor, the amount of pain medication needed, the need for emergency care for the baby, and improvement in maternal dietary diversity [31,45,46,47]. The present finding sought to indicate that these observations were also reported from other studies conducted in Malawi [48] and Bangladesh [49]. On the contrary, the findings of this study differ from that of central Malawi [45], where men were gradually becoming active in nutritional activities related to maternal and child health, such as providing food and in some cases even preparing food, which was previously a domain associated only with women.

Food insecurity is associated with a decreased nutritional status, including limitations in the quality, quantity, and/or frequency of food intake [50,51]. In line with previous findings from Ethiopia [36], households’ lack of adequate food and financial resources directly affected women’s abilities to follow healthy nutritional practices. Respondents attributed food and financial difficulties such as unemployment or under-employment of men/women, lack of farm land, and a burdened economic system to the poor inadequate intake of food during pregnancy. Most of the respondents agreed on the importance of nutrition during pregnancy and were generally aware that it has impacts on the health of the mother and the baby. However, a majority of them reported that food insecurity among pregnant women was the main barrier to consumption of a nutritious diet. The present study is consistent with studies conducted by Torheim [52] and Muzi [53], who reported that decline in maternal dietary diversity was positively associated with increasing severe household food insecurity; and women from the most food-insecure households were consistently and significantly less likely to report consuming animal-source foods.

A considerable proportion of the study participants still believed that some foods should not be eaten during pregnancy. While the reasons for the identified food taboos are not scientifically supported, they seem to have developed from the intention to prevent pregnancy complications for the mother and any health problem for the baby (Table 2). These results were consistent with similar earlier studies done in other regions of Ethiopia and India [36,54]. There is also a fasting season for pregnant women who follow the Orthodox religion, requiring that they do not eat animal foods during the fasting period (which spans more than 180 days per year). A similar finding from a study conducted in other parts of Ethiopia reported that there are 250 fasting days per year during which fasting Orthodox Christians abstain from consumption of any sort of animal foods [55]. However, according to Murphy [56], animal foods are an essential component of a nutritious diet, particularly for the health of pregnant mothers and their children. Similarly, an online cross sectional survey reported that avoiding or reducing animal source foods during pregnancy was found to be of major concern in young women [57]. Though none of them were mentioned in this study, many culturally informed prenatal food restrictions were related to ensuring that the baby (or his/her head) is not too big, in order to avert a difficult labor [42].

Unplanned investments in traditional and cultural ceremonies were reported as reasons for food shortages and hence nutritional problems in the most vulnerable segments of the population, particularly mothers and their children. The study findings are consistent with Rao [58], who reported that a typical household spends approximately seven times its annual income on a daughter’s marriage—celebrating the wedding and on dowry transfers to the groom’s family in rural India. Moreover, 15% of its expenditures, on average, is spent on celebrating village festivals. As the result, poor households tend to borrow large sums of money and eventually go into debt and drain household resources that could be destined towards improving maternal nutrition during pregnancy.

Our findings indicate that the coverage and quality of information provided to pregnant women was poor and this is inconsistent with a study that reported that nutrition information was a significant predictor of dietary practices in Ethiopian women [59]. The nutrition-related information provided during pregnancy or antenatal care visits is inadequate and more importantly not tailored towards the women’s dietary habits, cultural background, nutritional knowledge, and level of nutritional literacy [60]. A systematic literature review on sources of nutrition information accessed by pregnant women revealed that women were not receiving adequate nutrition information during pregnancy due to lack of time, lack of resources, and lack of relevant training of health professionals [61]. The poor physical access to health facilities due to geographical accessibility and limited health infrastructure are important barriers to health service utilization [62,63,64]. During the current study, participants reported that the lack of physical access to and low quality of health facilities was indicated as a barrier to pregnant women to utilize the health and nutrition services. Similarly, the lack of infrastructural development such as roads, lack of public transport and proximity to functional health facilities for rural residents remains a great problem in Nepal [65]. Similar findings were reported in Uganda [66], where distance to service points, perceived quality of care, and availability of drugs are key determinants of access to and utilization of health services. 

Adequate nutrition during pregnancy as part of the first 1000 days is critical to reducing the risk of adverse birth outcomes and improving the nutritional status of children and pregnant women [67]. However, our study findings indicate that more attention is given to child health and nutrition than to the health of pregnant women, by both the government and husbands. Similar findings were reported in Zambia [68]. A recent review of the evidence and program implications on maternal nutrition from low- and middle-income countries revealed that many programs still target the implementation and monitoring of nutrition interventions to infants and young children, rather than to women during pregnancy or post-partum, suggesting that monitoring of progress in maternal nutrition during pregnancy is still an area of concern to the nutrition community [69].

### Strengths and Limitations

The inclusion of different study groups to grasp the barriers and implementation challenges for optimal nutrition practices, along with collaborative development and pretesting of the data collection tools were strengths of this study. The use of a qualitative approach enabled us to collect deeper information particularly relevant to the study objectives. Furthermore, study participants were organized in two different groups of food in-secure and food secure areas, which allowed us to gain access to populations with different ways of economic and living standards. In addition, the inclusion of a sufficient number of participants allowed for saturation of information and hence breadth of knowledge.

The study had some limitations. Although the KIIs and FGDs were carefully conducted, we do not know to what extent the participants over-reported or underreported positive or negative behaviors. While the tabias/kebelles were randomly selected, they may not represent the whole population of the districts found in the study areas.

## 5. Conclusions

This qualitative study was conducted to explore the socio-cultural barriers and implementation challenges that limit the uptake of nutrition services among pregnant women from the rural communities of Northern Ethiopia. Maternal factors such as lack of awareness, low educational status, poor dietary habits, dependency syndrome; household factors such as heavy workload, poor husband support, lack of economic resources; community factors such as food taboos and avoidances, increased expenditure for cultural and religious festivities, limited sources of nutrition information; and health/nutrition service factors such as poor access to health facilities, poorly equipped health facilities, focus on child health and nutrition, poor coordination among nutrition specific and sensitive sectors were identified as the socio-cultural barriers and implementation challenges that limit the uptake of nutrition services during pregnancy. Considering the diversity of the socio-cultural barriers and implementation challenges for the poor uptake of nutrition services, a combined strategy of culturally sensitive nutrition education, social and behavior change communication and multi-sectoral coordination between the nutrition specific and nutrition sensitive sectors is recommended to improve nutrition during pregnancy in rural communities of Ethiopia.

## Figures and Tables

**Table 1:** Characteristics of the FGD and KII participants, Tigray, Northern Ethiopia, 2017/18.

Participant Characteristics	KIIs, *n* (%) *N* = 90	FGD Participants, *n* (%) *N* = 95
**Education**		
Formal education	-	63 (66.3)
Elementary and secondary education	18 (20)	32 (33.7)
College and above	72 (80)	-
**Occupation**		
Farmer	14 (15.6)	92 (96.8)
Government employee	76 (84.4)	3 (3.2)
**Distribution**		
Agriculture experts	10 (11.1)	-
District education heads and school teachers	13 (14.4)	-
Health extension workers	8 (8.9)	-
Health workers	10 (11.1)	
Husbands	--	3 (21.4)
Nutrition focal persons from the district and regional health offices	9 (10.0)	-
Pregnant mothers	6 (6.7)	8 (57.2)
Religious leaders	2 (2.2)	-
Tabia/kebelle leaders	7 (7.8)	-
Water resource experts	4 (4.4)	-
Women development groups	6 (6.7)	3 (21.4)
Women sector experts	9 (10.0)	-
Youth association heads	6 (6.7)	-
The average length of discussion in minutes *	57.5	80

* Data presented as Mean ± Standard Deviation.

**Table 2:** Food taboos and the reasons for their avoidance during pregnancy in the study communities, Tigray, Northern Ethiopia, 2017/18.

Serial Number	Food Taboos During Pregnancy	Reasons for Avoidance
1	Qollo (roasted) of chickpea and wheat	Causes abdominal cramp in newborns
2	Green pepper	Green pepper affects eye of the infant
3	Hot coffee	Causes balding in children
4	Senaficho (dressing made from brassica)	Could cause miscarriage
5	Alcohol	Affects the health of the baby.
6	Shiro (stew/sauce) made from legumes	Provides no calorie/energy and do not protect from anemia
7	Roasted beans (Qollo of roasted bean)	Causes pain during delivery
8	Millet	Consumption causes constipation in pregnant women
9	Niger oil	Consumption of Niger oil causes skin darkness (black color)
10	Pea, bean, and maize	Causes nausea during pregnancy

## Supplementary Materials

The following are available online at http://www.mdpi.com/2072-6643/10/11/1687/s1, Table S1: A sample of some of the questions posed to participants during the interviews and focus group discussions.

## Abbreviations

FGD: focus group discussions; KII, key informant in-depth interviews; IDI, in-depth interviews; NGO, non-government organization
