# Supplementary material for: Demand and Supply Side Barriers that Limit the Uptake of Nutrition Services among Pregnant Women from Rural Ethiopia: An Exploratory Qualitative Study
Source: Nutrients. 2018 Nov 5;10(11):1687. doi: 10.3390/nu10111687 (PMC6267174; doi:10.3390/nu10111687)
Supplement: Supplementary file 1 [file nutrients-10-01687-s001.pdf]

Appendix: Supplementary materials

Table S1. A sample of some of the questions posed to participants during the interviews and focus group discussions.

Serial Number	Questions
1	What are the common nutrition problems in the community for pregnant women?
2	What pregnant women nutrition interventions are the priorities in this community?
3	How do you perceive/evaluate the priority given for nutrition interventions during pregnancy?
4	What interventions for pregnant women are being implemented in an effective way?
5	What are the challenges to implement delivering nutrition interventions for pregnant women?
6	What are the perceived needs of nutrition services during pregnancy?
7	How aware and interested are the pregnant women on the need to utilize the nutrition interventions/services?
8	What community related beliefs and norms are preventing access to nutrition interventions?
9	How convenient are the nutrition services for pregnant women?
10	How do you judge the commitment of the nutrition services providers during pregnancy?
11	What would be the best way to get nutrition information to pregnant women?
12	Who should provide nutrition information during pregnancy? Why?
